# Has previous loan rejection scarred firms from applying for loans during Covid-19?

**DOI:** 10.1007/s11187-021-00586-2

**Published:** 2021-12-18

**Authors:** Marc Cowling, Weixi Liu, Raffaella Calabrese

**Affiliations:** 1 University of Derby, Kedlestone Road, Derby, DE22 1GB UK; 2 University of Bath, The Avenue, Claverton Down, Bath, BA2 7AY UK; 3 University of Edinburgh, Old College South Bridge, Edinburgh, EH8 9YL UK

**Keywords:** Scarred borrowers, Discouraged borrowers, Bank loans, SMEs, Covid-19, H81, L26, G21, G01, G4

## Abstract

**Abstract:**

The concept of the ‘discouraged’ borrower is well documented. In this paper, we consider whether smaller firms in the UK who have been previously rejected for bank loans have been scarred by the experience so badly that even in the presence of two exceptionally generous Covid-19 loan guarantee schemes, they still refuse to make an application. Furthermore, we also consider what happens when they do. As banks have either zero or minimal loss exposure, do they still maintain their normal strict lending protocols or do they relax their standards to fulfil the governments’ objective of supporting struggling businesses through the crisis? Our findings show that 72% of previously rejected borrowers are reluctant to request loans. We find some evidence that previously scarred firms faced such severe liquidity problems that they relaxed their distrust of banks during the Covid-19 crisis. However, their share of the government-guaranteed loan portfolio was slightly lower suggesting that banks were treating each new loan application on its merits.

**Plain English Summary:**

The Covid-19 crisis hit smaller businesses so hard that even previously rejected borrowers were forced to apply for loans to keep them afloat. Previous loan rejections have not discouraged small businesses in the UK in applying for Covid-19 government-guaranteed loans. Banks have used the loan guarantee schemes to continue to supply loans to small business during the pandemic. Our paper analyses the important phenomenon of borrower scarring and discouragement, when potential debtors are self-excluded from the lending market because they have previous rejections or expect a negative bank response. We consider around 45,000 UK small businesses from 2018 to 2020. On the demand side, we find that the economic shock for small businesses during the pandemic dissipates the scarring effect. Specifically, we find that micro and small businesses had the highest loan demand in the first two quarters of the pandemic (from March 2020). On the supply side, we show that scarred borrowers were not routed onto Covid-19 government-guaranteed loan schemes. These findings show the importance of government-backed lending schemes for small businesses during crisis period.

**Supplementary Information:**

The online version contains supplementary material available at 10.1007/s11187-021-00586-2.

## Introduction

The concept of the discouraged borrower is well documented (Kon & Storey, 2003) and has been identified as an important real-world phenomenon (Cole & Sokolyk, 2016; Cowling et al., 2016; Fraser, 2009). For the main however, it is framed in a rather static way as certain groups of potential borrowers self-exclude from the loan market on the basis of their *perceptions* of a negative bank response. In this paper, we extend this framing to include potential borrowers who have had previous loan application rejections and are ‘scarred’ by this negative experience so much that they self-exclude from the loan market after that point. In this sense, we argue that the actual reality of being refused a loan adds more tangibility to the decision to self-exclude as it is based on lived experience rather than perception. We term this group *scarred borrowers*. Furthermore, having identified the extent and nature of scarred borrowers, we overlay their current financing behaviours with the Covid-19 crisis and question whether the sheer severity and scale of the crisis have encouraged scarred borrowers to change their behaviours.

Scarring is a well-understood concept in Labour Economics where scarring from lengthy periods of unemployment has been found to be an important characteristic that also has very real and negative economic effects (Arulampalam, 2001), particularly during ‘Great Recessions’ and immediately afterward (Tumino, 2015). It has also been used as to describe the potential long-run legacy of the Covid-19 crisis. On this, Kozlowski et al., (2020: page 1) argue that the crisis has caused a scarring of beliefs, which they define as a ‘persistent change in the perceived probability of an extreme, negative shock in the future’. It is this aspect of this latter concept that is most relevant in this study given the current debates about what the post-Covid-19 recovery might look like and how long it might take firms to return to their steady-state patterns of savings, borrowing and investment. Our argument is that we need to establish whether Covid-19 itself changed established firm behaviours during the crisis before we can predict the potential longer run impacts.

The lens that we look through in this paper is the small firm debt market. We analyse 10 waves of cross-sectional data on 45,000 UK SMEs from the second quarter in 2018 to the third quarter in 2020. Our primary research question is whether scarring due to pre-Covid-19 loan application rejections has prevented struggling small firms from putting forward loan applications even in the presence of the two most generous ever government-backed loan guarantee programmes (CBILS, the Coronavirus Business Interruption Loan; and BBLS, the Bounce Back Loan Scheme) which offer a guarantee coverage of 80% and 100%, respectively, to the lending bank. In this sense, we are questioning how bank loan rejections in the ‘good’ times pre-Covid-19 might have permanently scarred small businesses to the extent that even during Covid-19, when trading activities were forcibly restricted, and many firms faced significant liquidity and cash flow pressures, they were more reluctant to put forward loan applications.

We then consider the loan supply side of the small firms’ debt market and question whether banks have relaxed their lending standards in the presence of the two generous loan guarantee schemes. Previous research highlights the fact that the incidence of financial delinquency (e.g. unauthorised overdraft limit transgressions, legal non-payment of debt obligations, late payment of taxes, etc.) is a fundamental determinant of the banks’ lending decision-making process (Cowling et al., 2016). Thus, if previous loan application rejections informed their historical rejections, then it follows that banks viewed these firms as bad credit risks at that time. The current state of the world and particularly the presence of a zero or 20% lending risk may have encouraged banks to lower their normal lending standards in this respect which would result in an increased willingness to lend during the Covid-19 crisis.

The rest of the paper is organised as follows: in Section 2, we review the literatures on small firm debt markets and the loan contracting process. We draw in the related concepts of the discouraged borrower and consider the importance of an established pecking order or financing hierarchy. We also present a theoretical model for scarred borrowers. In Section 3, we present our data, discuss the sample demographics and outline our analytical methodology for exploring our fundamental research question. Section 4 presents our core modelling results, and we conclude in Section 5.

## Literature review

In this section, we draw from a broad literature relating to small firm debt markets and loan contracting with a particular focus on a financial pecking order and the presence of borrower discouragement. Prior to that, we consider the literature around negative economic shocks and how they might influence beliefs about the future potential for extreme events to occur and the impact if they do. This has relevance both to the concept of scarring and the perception that a negative future event will occur.

### Negative economic shocks

The UK Brexit vote in June 2016 delivered an unexpected outcome (the vote to leave the European Union) and the intervening 4 years until the Covid-19 crisis hit was one of uncertainty and low borrowing and investment. In its’ own right, Brexit could be seen as an extreme event which altered behaviours. Ten months before the final exit from the European Union in January 2021, the Covid-19 crisis unfolded another extreme, and unanticipated, event. And this ignores the global financial crisis (GFC) that occurred in late 2008 and persisted until 2011 in the UK during which banks severely rationed credit to smaller firms with loan application acceptance reaching a nadir of 65% (Cowling et al., 2012). In *extremis*, some small business owners, whose banks were happy to lend to prior to GFC who then faced rejection during GFC, may have downwardly revised their expectations of a positive loan outcome and become scarred at this point, whilst some may have become scarred on an individual basis through a simple loan rejection at any time since. Taken overall, most small business owners will know two things that might shape their view on the world: firstly, that banks do not treat them well during a crisis (Udell, 2020), and secondly, that crises happen on a quite regular basis. But the precise cause and severity of each crisis are different and hence any learning is non-parametric as it is derived from the tails of the risk belief distribution (Kozlowski et al., 2020).

The most recent events that generate financial turmoil that had a significant impact on the credit availability for SMEs in the UK are the GFC, Brexit and the Covid pandemic. The literature on the effects of the GFC on access to finance for SMEs is extensive (Cowling et al., 2012; Popov & Udell, 2012; Udell, 2020 amongst others), but the number of empirical studies on the impact of Brexit is still limited (Calabrese et al., 2020; Brown et al., 2019). This number is even lower for the Covid crisis because it is a recent event and, therefore, limited data is available (Cowling et al., 2021). One of the aims of this paper is to fill this gap in the literature and provide some empirical evidence on the Covid crisis.

Negative economic shocks could have different effects on the demand for external finance. On the one hand, it is likely that loan demand declines when bank capital decreases, such as during the GFC, because SMEs are usually discouraged given the high number of unsuccessful loan applications in such periods (Cowling et al., 2016). On the other hand, several empirical studies showed that the demand for external finance increases during economic downturn (Bank of England, 1993; Binks et al., 1992). Specifically, Cowling et al., (2012) and Popov and Udell (2012) obtained that as profits decrease and cash flow tightens, firms are more likely to seek for external finance during the GFC. This evidence gives rise to our first hypothesis:

H1: The general demand for loans will increase during Covid-19

Negative economic shocks usually generate an increase in uncertainty that exacerbates information asymmetries between borrowers and lenders (Mishkin, 2011; Sette & Gobbi, 2015; Jappelli and Pagano, 2002). Consequently, banks tend to cut the credit supply during such periods toward small businesses (Cowling et al., 2012). We therefore examine whether the supply of bank loans to small businesses decreases during Covid-19 compared, so our second hypothesis is as follows:

H2: The willingness of banks to supply conventional loans will decrease during Covid-19

### Debt markets and SME loan contracting

Bank loans are the most prevalent and important form of external finance for smaller firms (Carpenter & Petersen, 2002). This in part relates to the relative unsophistication of the typical small business owner in respect of financial acumen and knowledge of more complex financial instruments, and also to the ease of the application process and the speed of the bank decision-making process (Howorth, 2001). The key characteristic of the process of loan contracting between small firms and banks is the presence of asymmetric information—one agent in the contract having superior information about the true quality of the lending proposal than the other (Stiglitz & Weiss, 1981). This information advantage is usually assumed to be in favour of the entrepreneur, although some researchers have argued that a large banking group has millions of small business accounts and a history of repeated loan transactions and that this gives it an informational advantage over the entrepreneur (De Meza, 2002). Given the primacy of bank loans in meeting the external financing needs of smaller businesses and the offer of a government-guaranteed loan, we hypothesise that loan demand and supply will increase during the Covid-19 crisis.

### Pecking order hypothesis

The pecking order hypothesis (Myers & Majluf, 1984) states that firms will have a financial pecking order within which internally generated funds will be preferred to external debt, and external debt will be preferred to equity as that involves a legal claim on the ownership of the firm and future stream of profits, thus raising independence and control aversion issues. This is a combined outcome of the personal preferences of the small business owner and also information-based capital constraints in external markets where insiders have a more perfect knowledge about the true value of a project, and the firms’ prospects, than outsiders (Watson & Wilson, 2002). It follows that any variation in debt financing will be a direct result of a decline in internal funds (Shyam-Sunder & Myers, 1999). Problems with internal cash flows are related with a firms’ investment opportunity set. A firm can be profitable but have limited investment opportunities leading to a big cash pile and low debt levels. Equally, a firm can have lots of profitable investment opportunities but have exhausted internal cash which triggers higher borrowing.

The high general levels of non-borrowing amongst the small firm population suggest that in normal circumstances, the majority of small firms would self-finance their operational and investment needs. In the Covid-19 crisis, recent research has identified a lack of precautionary saving and retained earnings amongst micro and small businesses and this has directly affected their liquidity position during the series of business lockdowns that were imposed by government (Brown & Cowling, 2021; Cowling, Brown, et al., 2020; Cowling, Liu, et al., 2020; Cowling, Nadeem, et al., 2020). This lack of internal funds would imply that more firms would move to their second preference which is bank loans to shore up their cash position even in the face of declining opportunities for profitable investment.

### Discouraged borrowers

The seminal work of Jappelli (1990) identified as liquidity-constrained, borrowers who were either discouraged or turned down for credit, defined as denied firms. Cole and Sokolyk (2016) showed that discouraged and denied firms are significantly different in terms of size, profitability and the number of sources of financial services.

Given this difference, Kon and Storey (2003) defined a theoretical model only for discouraged borrowers, defined as businesses who have a demand for capital but do not translate that demand into a formal application for bank loans. In the presence of imperfect information in small firm debt markets, banks incur screening costs and importantly these costs are passed on to the borrower. This raises the application costs to the small firm which makes some potential projects unviable as they are unable to make the normal returns required to pay capital and interest plus screening costs. Furthermore, banks make screening errors, thus rejecting ‘good’ risk loan applications. It follows that the higher the screening costs imposed by the banks and the greater the screening error, the higher the level of borrower discouragement.

A subsequent body of empirical measurement, quantification and testing of discouraged borrowers established that it is a genuine phenomenon amongst the small business population and quantifiably a non-trivial subset of smaller business populations across the world (Cowling et al., 2016; Han et al., 2009). Some studies (Cole & Sokolyk, 2016; Freel et al., 2012) show that discouraged borrowers can outnumber actual turndowns. Other work has focused on ethnicity (Fraser, 2009), gender (Freel et al., 2012), Eurozone economies (Mac an Bhaird et al., 2016), the role of bank-firm trust (Tang et al. 2017) and the potential for political connections to influence banks’ decision-making (Qi & Nguyen, 2021). Importantly, estimates from this body of empirical work also suggest that between one third and one half of discouraged borrowers would have been offered a loan had they applied. This suggests that self-rationing is often based on poor assumptions.

In the context of the Covid-19 crisis, we would expect that greater general uncertainty and higher levels of asymmetric information would increase the costs of screening and discourage more firms from making loan applications. However, this would potentially be mitigated by the presence of the government-backed guaranteed lending schemes as screening to reduce lending risk is less essential when the government guarantee reduces lending risk and loss given default significantly.

### Borrower scarring

In respect of a loan application outcome, the small business owner has a view on the potential distribution of outcomes which is informed by her previous history of loan applications. This initial view on the outcome distribution might be revised in the light of a loan application event, particularly if it generates a less favourable outcome than expected. Some empirical studies showed that prior loan rejection often discourages a firm from applying again in the future (Cavalluzzo et al., 2002; Xiang et al., 2015). Therefore, we define the *scarred borrower*, who, in the face of an unexpected loan rejection, is so scarred by the experience that this fundamentally reshapes their view on the distribution of future applications outcomes that they become reluctant to make future applications. This means that scarred borrowers are the subset of discouraged borrowers that have been previously turned down for credit.

In line with Cowling et al., (2012) and the previous studies on prior loan rejections, we propose the following hypothesis:

H3: The deeper into the Covid-19 crisis we go, the higher the demand for loans due to cash flow pressure on firms and formerly scarred borrowers (through choice) changing their behaviours

As the credit supply decreases during severe recessions, such as the early thirties in the USA (Welsh, 1952) and during the Covid pandemic, the government can provide an increment to the supply of credit through loan guarantee schemes. Given the unprecedented effects of the Covid-19 pandemic on firms, the UK government supported the capitalisation of businesses through the CBILS and the BBLS. If a loan guarantee scheme is effective, most of the firms obtaining bank loans through such a scheme were unlikely to obtain financing from traditional sources (Cowling & Mitchell, 2003; Riding et al., 2007). We expect that the more severe the economic downturn of the Covid crisis, the lower are firm profits and weaker is their cash flow position, so the higher is their demand for external finance in general (Cowling et al., 2012) and for the government-guaranteed loans, as our final hypothesis states:

H4: The deeper into the Covid-19 crisis we go, the higher the use of government-guaranteed loans to meet the higher demand of credit

### Theoretical model for scarred borrowers

Following Kon and Storey (2003), we propose in this section a theoretical model for scarred borrowers that are discouraged by applying for external finance given a previous loan rejection. We assume that there are two types of firms, good (G) and bad (B), and each firm needs one unit of funding for its investment that it can borrow from a bank or from an alternative source, called money lender. We also consider the 10 assumptions reported in Kon and Storey (2003) and that a bank can correctly identify good and bad firms. Under these assumptions, the expected return from applying is

1 $$\left(1-{\mathrm{p}}_{\mathrm{b}}\right) \left({\mathrm{X}}_{\mathrm{G}}-\mathrm{D}-\mathrm{K}\right)+{\mathrm{p}}_{\mathrm{b}}\left(\mathrm{w}-\mathrm{K}\right)$$

where

- *X*_G_—gross return from the project funded by the loan
- *D*—interest rate charged on the loan
- *K*—application costs for the loan
- *w*—net return of the project funded through the money lender after interest payment
- *p*_b_—probability that the firm assesses itself as bad

**Fig. 1:**
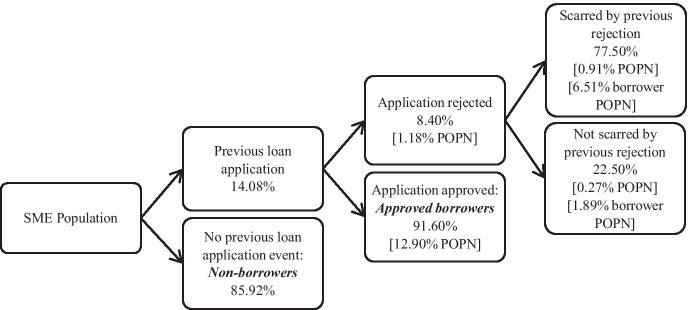
The process of (pre-Covid-19) historical borrower scarring

To compute the probability *p*_b_, we classify all the borrowers as firms with no previous loan applications, borrowers whose previous application was successful and scarred and unscarred rejected borrowers, as displayed in Fig. 1. Based on the law of total probability, we can compute *p*_b_ as follows:

2 $${\mathrm{p}}_{\mathrm{b}}={\mathrm{p}}_{\mathrm{b}1}\bullet \mathrm{p}\left(\mathrm{NPA}\right)+{\mathrm{p}}_{\mathrm{b}2}\bullet \mathrm{p}\left(\mathrm{APA}\right)+{\mathrm{p}}_{\mathrm{b}3}\bullet \left[1-\mathrm{p}\left(\mathrm{APA}\right)-\mathrm{p}\left(\mathrm{NPA}\right)\right]$$

where

- *p*(NPA)—the probability of not having a previous history of loan application
- *p*(APA)—the probability of having a previous loan application approved
- *p*_b1_—probability that the firm assesses itself as Bad given that the firm did not previously apply for a bank loan
- *p*_b2_—probability that the firm assesses itself as Bad given that the firm had a previous application approved
- *p*_b3_—probability that the firm assesses itself as Bad given that the firm had a previous application rejected.

Figure 1 shows that p(NPA) = 0.8592 and p(APA) = 0.1290.

If the expected return for non-application is *w*, a firm will apply for loan if the following condition is satisfied

3 $$\left(1-{\mathrm{p}}_{\mathrm{b}}\right) \left({\mathrm{X}}_{\mathrm{G}}-\mathrm{D}-\mathrm{K}\right)+{\mathrm{p}}_{\mathrm{b}}\left(\mathrm{w}-\mathrm{K}\right)>\mathrm{w}$$

To incorporate the empirical evidence that prior loan rejection can discourage a company from applying in the future (Cavalluzzo et al., 2002; Xiang et al., 2015), we assume in our model that *p*_b3_ > *p*_b2_.

## Data, sample demographics and methodology

In this section, we discuss the source of the data which we used for our empirical analysis of the potential impact of previous loan rejection, scarring and how these impacts on loan demand and supply during the Covid-19 crisis. We then discuss our methodology for identifying these issues in the context of firms making a loan application, the banks’ decision and whether successful applications were routed through the two UK government loan guarantee schemes.

### The SME Finance Monitor

The data we have available for analysis is the most recent ten waves of the SME Finance Monitor (SFM) covering in total a cross-section of 45,018 individual firm-level observations and the period from 2018 quarter 2 and 2020 quarter 3. The last two waves are the SFM special Covid-19 survey for the period April to September 2020. These specific survey waves are unique within the context of the 36 SFM waves that preceded them as they incorporated some specific questions relating to the UK government Covid-19 lending schemes (CBILS and BBLS). It is this additional information that we use to tackle our key research questions.

Because of their low natural representation in the SME population, larger SMEs are over-sampled in the SFM survey using the classic B2B sample structure in order to generate robust sub-samples of these bigger SMEs. In particular, the SFM sample includes a pre-determined percentage of SMEs (quota) by size: 20% zero-employee, 33% micro (1–9 employees), 32% small (10–49 employees) and 32% medium (50–249 employees) firms. Additional quotas were set by sector and region, which were then allocated within employee size band to ensure that SMEs of all sizes were interviewed in each sector and region. To further qualify for the SFM interview, SMEs had to meet three criteria in addition to the quotas by size, sector and region: (i) not 50% + owned by another company; (ii) not run as a social enterprise or as a not-for-profit organisation; and (iii) turnover of less than £25 m. Each quarter’s sample matched the previous quarter’s results as closely as possible.

Each of the 5,000 respondent firms interviewed per wave (by CATI using the quota sampling method) is allocated a Dun & Bradstreet and Experian credit risk rating and this is added to the data set on a case-by-case basis. Sample weights were applied based on the above three strata—size, sector and region—and then for firms trading for fewer than 2 years (start-ups). Weights were initially applied separately to each wave and both waves were then combined and grossed to the total of 5,002,010 SMEs, based on UK SME population data. This ensured that each individual wave is representative of all SMEs whilst the total interviews conducted are weighted to the total of all SMEs. After eliminating missing values,^1^ we have 30,577 valid observations across ten SFM waves.

The key dependent variables that link to our research questions are (i) *sought loan*, coded 1 if firm sought a bank loan during the Covid pandemic (Q2 and Q3 2020) and 0 if not; (ii) *got loan offer*, conditional on applying for a loan in the first instance, coded 1 if the firm received an offer from the bank and 0 if refused; and (iii) *government-backed loan offer*, conditional upon applying for a loan and receiving an offer, coded 1 if firms offer was a government-backed loan and 0 if not (i.e. a conventional bank loan).

Prior to our examination of the Covid-19 loan demand and supply, and the use of the UK government loan guarantee schemes, we need to go further back into survey time to the pre-Covid-19 period to define scarring in the context of previous loan application rejections. The key survey questions that we use for this preliminary classification of scarred and non-scarred loan applicants and approved applicants are the following:

[Scarred Borrower Q1]: Thinking more broadly, has the business EVER had either an application (for a loan) rejected or declined? (response, Yes/No)

[Scarred Borrower Q2]: And has this previous (loan) decline made you more reluctant to apply for another loan? (response, Previous decline has made more reluctant to apply/*Previous decline has NOT made more reluctant)

### Summary statistics

Figure 1 in Section 2.6 graphically represents the step-by-step categorisation of our scarred and unscarred rejected borrowers, successful borrowers and firms with no history of making loan applications. We observe that overall 85.9% of firms had no loan applications leaving a potential lending pool of 14.1% of smaller businesses seeking loans. Of these loan applicants, the approval rate was 91.6% which is in line with other UK evidence on banks’ willingness to supply loans to smaller businesses (Cowling et al., 2020a,b). The fact that the vast majority of the small business population do not make loan applications confirms the empirical validity of the pecking order hypothesis (Myers, 1984; Myers & Majluf, 1984) which established those smaller businesses will also have a stronger preference for financing their operations and investment activities from internal funds whenever possible (Michaelas et al., 1999; Revest & Sapio, 2012).

Of the subset of the population that had been refused a loan prior to the Covid-19 crisis, 72.4% reported that they were now reluctant to make further loan applications. We assign this group of potential borrowers as *scarred borrowers*. Those 27.6% of firms who were rejected but reported that they remained willing to make further loan applications in the future are classified as *non-scarred borrowers*. Thus, *scarred borrowers* make up 6.1% of the total pool of potential lending pool, *unscarred borrowers* 2.3% and *successful borrowers* the remaining 91.6%. In this sense, *scarred borrowers* are a small but potentially important segment of the small firm potential borrowing population, although only 0.85% of the total small business population overall which is dominated by firms that do not seek external finance (85.9% of the total small business population).

**Fig. 2:**
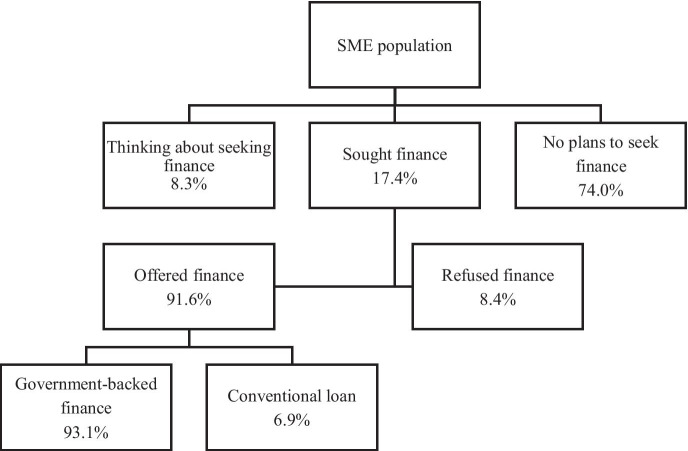
The demand and supply of bank loans during Covid-19

Figure 2 shows the loan demand and supply during the Covid-19 crisis when the two government guarantee schemes (CBILS and BBLS) were in place. We observe slightly higher loan application rates of 19.3% (up from 14.1% pre-Covid-19) and identical loan acceptance and refusal rates. An interesting feature was that a much smaller share of the small business population had no plans for borrowing than the permanent non-borrowers identified in the pre-Covid-19 period (74.0% during Covid compared to 85.9% pre-Covid). This suggests that sheer magnitude of the Covid-19 crisis and its impact on smaller firms had weakened the resolve of many businesses that have explicitly sought to avoid taking on debt.

The absolutely striking feature of the debt market landscape during the Covid-19 period was that 92% of all issued loans were offered through the government guarantee schemes. This compares to a maximum of 3% of total lending in normal pre-Covid-19 circumstances (Cowling, 2010). Thus, banks are willing to commit loan funds during the crisis due to the size of the guarantee which covers 80% of CBILS lending and 100% of BBLS lending. The interesting subset of the 7.9% of firms who made a successful application and were offered a conventional (non-government-backed loan) suggests that the bank sought to lend to preferred customer firms outside of the scheme where it had the ability to set different contractual terms given both schemes had in-built repayment holidays and a fixed rate of interest.

Table 1 reports the definitions and summary statistics of all dependent and independent variables. The independent variables capture common business characteristics, firm-level risk indicators and firm orientations. Firm characteristics include sales, employee number, legal status, sector, firm age and performance. Performance is measured by firstly a (made a positive) profit dummy variable, secondly the banded percentage sales growth and thirdly a fast-growth variable. We use Experian risk classification to measure credit risk from minimum to above average. Firm orientation is proxied by two variables: the owner-manager’s aims to grow the business (*AIMGROW*), and their perception of growth obstacle (*FINPROBLEM*). We also include a control for firm-bank relationship.

**Table 1:** Variable definition and descriptive statistics (weighted; *N* = 30,577*)

Variable	Definition	Full sample		(1) SOUGHT = 1	(2) SOUGHT = 0	*T* test	(3) GOT = 1	(4) GOT = 0	*T* test	(5) GOT_GO*V* = 1	(6) GOT_GO*V* = 0	*T* test
		Mean	Std. Dev	Mean	Mean	(1) = (2)	Mean	Mean	(3) = (4)	Mean	Mean	(5) = (6)
Dependent variables												
SOUGHT	(Q2&3 2020 only) = 1 if firm applied for loan; 0 otherwise	0.193	0.394									
GOT	(Q2&3 2020 only) = 1 if firm offered the finance applied for; 0 otherwise	0.925	0.263									
GOT_GOVT	(Q2&3 2020 only) = 1 if firm’s offer was a gov’t-backed loan; 0 otherwise	0.921	0.269									
Historical rejection	= 1 if firm historically rejected loan; 0 otherwise	0.037	0.190	0.094	0.033	***	0.089	0.337	***	0.080	0.237	
Scarred by rejection	= 1 if non-applying due to historical rejection; 0 otherwise	0.724	0.447	0.793	0.711		0.733	0.964	***	0.752	0.636	
Independent variables												
Sales	Annual sales turnover (£ 'Mil) of the past year	0.285	1.262	0.415	0.255	***	0.414	0.370	***	0.374	1.009	
Employment size	Dummy variables: number of employees *excluding* owner											
0	Zero-employee	0.764	0.425	0.660	0.783	***	0.666	0.639		0.666	0.712	
1–9	Micro	0.192	0.394	0.271	0.183	***	0.267	0.313		0.269	0.204	
10–49	Small	0.039	0.192	0.063	0.031	***	0.062	0.042		0.060	0.080	
50–99	Small-medium	0.004	0.062	0.005	0.003	***	0.004	0.003		0.004	0.003	
100–199	Medium	0.001	0.036	0.001	0.001		0.001	0.002		0.001	0.001	
201–249	Large-medium	0.000	0.018	0.000	0.000							
Firm age	Dummy variable: time when firm was first established											
< 1 year	Start-up	0.049	0.215	0.079	0.066		0.075	0.093		0.080	0.020	**
1–2 years	New	0.156	0.363	0.165	0.124	*	0.171	0.076	**	0.167	0.196	
2–5 years	Early stage	0.145	0.352	0.193	0.133	**	0.195	0.209		0.211	0.010	***
6–9 years	Established	0.133	0.340	0.126	0.132		0.131	0.187		0.133	0.106	
10–15 years	Well established	0.156	0.363	0.145	0.182	*	0.139	0.172		0.137	0.122	
> 15 years	Old	0.361	0.480	0.292	0.363	***	0.290	0.263		0.273	0.546	**
Legal	Dummy variables: legal form of the firm											
Sole proprietor		0.614	0.487	0.436	0.611	***	0.405	0.552		0.414	0.245	
Partnership		0.035	0.185	0.032	0.034		0.032	0.039		0.033	0.018	
LLP		0.020	0.139	0.004	0.005		0.003			0.003		
LTD Co		0.331	0.471	0.529	0.351	***	0.560	0.408		0.550	0.738	*
Industry	Dummy variables: industry sector											
Agriculture		0.030	0.170	0.018	0.034	*	0.018	0.019		0.019	0.004	***
Manufacturing		0.055	0.228	0.039	0.059	**	0.033	0.066		0.028	0.059	
Construction		0.189	0.392	0.152	0.197	**	0.146	0.199		0.157	0.019	***
Wholesale/retail		0.100	0.300	0.133	0.094	**	0.146	0.057	**	0.150	0.124	
Hotels and catering		0.034	0.182	0.047	0.033	**	0.051	0.035		0.050	0.066	
Transport and Comms	0.125	0.330	0.171	0.111	**	0.158	0.370	*	0.156	0.175		
Real estate		0.265	0.441	0.275	0.264		0.281	0.199		0.277	0.373	
Health		0.074	0.262	0.051	0.078	**	0.055	0.009	***	0.059	0.013	***
Other services		0.128	0.334	0.113	0.130		0.111	0.046	**	0.103	0.166	
Region	Dummy variable: geographic region											
Scotland		0.058	0.235	0.072	0.054		0.066	0.070		0.059	0.183	***
North East		0.029	0.168	0.030	0.031		0.020	0.008		0.017	0.061	
York and Humber		0.071	0.256	0.088	0.069		0.090	0.126		0.095	0.046	
North West		0.098	0.298	0.106	0.097		0.102	0.116		0.106	0.041	*
West Midlands		0.071	0.257	0.091	0.068		0.096	0.112		0.095	0.084	
East Midlands		0.070	0.255	0.083	0.069		0.094	0.065		0.102	0.002	***
East England		0.099	0.299	0.067	0.106	**	0.059	0.180		0.056	0.099	
Wales		0.041	0.198	0.045	0.042		0.043	0.045		0.038	0.046	
South West		0.100	0.300	0.085	0.109		0.086	0.025	***	0.090	0.040	
London		0.179	0.384	0.178	0.173		0.190	0.145		0.184	0.296	
South East		0.163	0.369	0.134	0.162		0.139	0.064	**	0.144	0.060	**
N. Ireland		0.020	0.141	0.020	0.020		0.017	0.044		0.015	0.042	
Risk rating	Dummy variables: Experian risk rating											
Minimal		0.058	0.234	0.058	0.053		0.064	0.070		0.049	0.263	
Low		0.136	0.343	0.144	0.120		0.133	0.125		0.137	0.088	
Average		0.278	0.448	0.233	0.279	*	0.241	0.222		0.245	0.205	
Above average		0.398	0.489	0.454	0.427		0.455	0.435		0.455	0.444	
Not known		0.130	0.336	0.111	0.121		0.106	0.149		0.114		
*PROFIT*	= 1 if firm made a surplus over the past year; 0 otherwise	0.742	0.438	0.702	0.717		0.706	0.655		0.724	0.403	**
FASTGROW	= 1 sales growth ≥ 20% by two consecutive years; 0 otherwise	0.034	0.181	0.073	0.026	***	0.072	0.023		0.077	0.005	
AIMGROW	= 1 if firm aimed to grow the business; 0 otherwise	0.464	0.499	0.420	0.293	***	0.417	0.371		0.423	0.332	
FINPROBLEM	= 1 if firm saw cash flow and/or external finance as main growth obstacle; 0 otherwise	0.143	0.350	0.248	0.138	***	0.220	0.300		0.228	0.149	
Sales growth	Growth of sales over the past 12 months											
> 40%		0.017	0.131	0.019	0.016		0.022	0.002		0.023	0.009	
20 to 40%		0.070	0.256	0.089	0.055	*	0.092	0.039	**	0.097	0.014	***
0 to 20%		0.192	0.394	0.148	0.152		0.138	0.149		0.134	0.221	
No growth		0.548	0.498	0.405	0.505	***	0.400	0.311		0.389	0.487	
< 0%		0.172	0.378	0.338	0.272	**	0.349	0.499		0.357	0.269	
Banking relationship	Strength of relationship with main bank											
Strong		0.216	0.411	0.243	0.189	**	0.260	0.118	*	0.261	0.210	
Fair		0.654	0.476	0.572	0.676	***	0.571	0.738	**	0.572	0.567	
None		0.130	0.336	0.186	0.135	**	0.169	0.144		0.167	0.223	

Except for *SOUGHT* (*N* = 5,052 and only observed in Q2 and Q3 2020 waves), *GOT* (*N* = 1,026 and only observed if SOUGHT = 1), and *GOT_GOVT* (*N* = 867 and only observed if SOUGHT = 1)

As shown in Table 1, the firm size distribution suggests that zero-employee firms are the dominant size class of firm, and of employer firms, micro businesses account for the vast majority. The median firm would be 10–15 years old with nearly four in ten very old, but there are significant minorities of new and early-stage businesses. At the industry level, real estate, renting and business services firms represent the single largest group of businesses, although there are also large shares in construction. On credit risk, we find that very few smaller firms would be considered minimal risk, and quite small shares as low risk. In contrast, 4 in 10 smaller businesses would be classed as above average risk and a non-trivial share has no formal risk classification per se. This group of firms would contain those who have never had a borrowing event or any incidence of financial delinquency (for example, late payment of a tax bill), and also those that conduct their businesses through their personal banking accounts or in cash. In terms of performance, although nearly three quarters of SMEs had made a profit in the previous year, the majority (73%) reported zero or negative sales growth and only a minimal proportion (3.4%) sustained high growth.

We then conduct three sets of univariate mean-comparison tests, for finance applicants vs. non-applicants, approval vs. rejection and recipients of government- vs. non-government-backed loans, respectively. It is shown that SMEs experiencing previous rejection were more likely to apply for loans during the pandemic. Within-Covid loan rejection in general was higher for both historically rejected and scarred borrowers, but such difference disappeared for government-backed loans. It is also found that non-applicants on average were smaller (both in sales and employment), grew more slowly and had higher credit risk, which lend support to the discouraged borrower literature that lower expected (or perceived) approval probability would deter firms from submitting loan applications. Furthermore, SMEs with higher growth orientations were more likely to seek finance. There is some, but limited, evidence that government-backed finance was targeting at most deprived businesses (firms less than 5 years old) and regions (such as North West and Midlands). We also report the summary statistics by region and industry in the online Appendix (Table A1) to highlight potential location- and sector-driven difference in financing activities.

### Methodology

By definition, scarring only happens after previous loan rejection, and loan approval is only observed for loan applicants. If we are to examine the effect of scarring on loan application and application outcome, the selection mechanism can be more than one layer. In particular, when sample selection is based on historical rejection only, in the case of (a) scarring from previous rejection and (b) the effect of scarring on Covid-19 loan application, we apply the Heckman (1979) two-step procedure through the inclusion of an inverse Mills ratio (IMR) from the loan rejection equation (step 1) in the outcome equation (step 2). In the case of the effect of scarring on loan acceptance, sample selection arises first from previous rejection and then current loan application. Similar is the offer of Covid-19 government-guaranteed loans, where there is one additional layer of selection mechanism—the acceptance of Covid-19 loan application. In the last two cases, we estimate the selection process through a multi-variate probit model in a sequential equation system, assuming correlated and jointly normal distributions of errors between each individual selection equations. We explicitly account for the process of selection through the sequence of equations by calculating the IMR for each equation, which enters the next step, outcome equation (acceptance of loan or government-guaranteed loan, respectively) as explanatory variable. So that the model is correctly specified, each selection equation should include a vector of variables (exclusion restrictions) that is not included in the outcome equation, which will be discussed in detail with individual specifications.

## Results

Here, we present the multi-variate regression results from our models relating to (a) previous incidences of loan rejection, (b) scarring effects of previous rejection, (c) current loan demand, (d) current loan supply and (e) whether loan offers used the UK government Covid-19 guarantee schemes. The models we use are pooled probit models allowing for the potential presence of sample selection effects as discussed in the previous section. All the regressions are population weighted and Table 2,^2^ reports the coefficient estimates as well as the marginal effects to show their economic significance.

**Table 2:** Regression results (population weighted)

Variables	(1) Historical loan rejection (probit)		(2) Scarred by rejection (probit with sample selection correction)		(3) Covid-19 loan application (probit with sample selection correction)		(4) Covid-19 loan acceptance (*GOT*) (probit with sample selection correction)		(5) Covid-19 government guarantee (*GOT_GOVT*) (probit with sample selection correction)	
	Coeff	dy/dx	Coeff	dy/dx	Coeff	dy/dx	Coeff	dy/dx	Coeff	dy/dx
*Scarred by rejection*					0.667***	0.159	− 1.317***	− 0.143	− 0.522	− 0.048
					(0.189)		(0.249)		(0.454)	
*Survey wave* (base: Q2 2018)										
Q3 2018	0.023	0.002	0.177	0.054						
	(0.104)		(0.310)							
Q4 2018	0.088	0.008	0.274	0.081						
	(0.111)		(0.303)							
Q1 2019	− 0.160	− 0.012	0.425	0.121						
	(0.115)		(0.333)							
Q2 2019	− 0.035	− 0.003	0.110	0.034						
	(0.106)		(0.293)							
Q3 2019	− 0.059	− 0.005	− 0.122	− 0.039						
	(0.113)		(0.324)							
Q4 2019	− 0.169	− 0.012	0.626*	0.168						
	(0.109)		(0.331)							
Q1 2020	− 0.086	− 0.007	0.293	0.086						
	(0.110)		(0.294)							
Q2 2020	0.052	0.005	0.467	0.131						
	(0.107)		(0.292)							
Q3 2020	− 0.014	− 0.001	0.181	0.055	0.257***	0.060	0.357	0.044	0.293	0.028
	(0.108)		(0.309)		(0.080)		(0.231)		(0.303)	
ln(s*ales*)	0.010	0.001	0.019	0.005	0.254***	0.060	0.409***	0.044	− 0.353	− 0.032
	(0.022)		(0.076)		(0.037)		(0.124)		(0.276)	
*Employment size* (base: 1 employee)										
1–9	0.117**	0.010	− 0.470***	− 0.139	− 0.148	− 0.034	− 0.538**	− 0.065	0.793*	0.072
	(0.057)		(0.163)		(0.097)		(0.249)		(0.469)	
10–49	− 0.000	0.000	− 0.638**	− 0.194	− 0.217	− 0.049	− 1.145***	− 0.187	1.049	0.085
	(0.087)		(0.249)		(0.144)		(0.335)		(0.737)	
50–99	− 0.182	− 0.012	− 1.425***	− 0.453	− 0.352*	− 0.074	− 1.824***	− 0.375	1.956*	0.110
	(0.123)		(0.392)		(0.198)		(0.494)		(1.058)	
100–199	− 0.213	− 0.014	− 1.069**	− 0.340	− 0.826***	− 0.141	− 3.400***	− 0.795	3.125	0.116
	(0.156)		(0.490)		(0.263)		(0.769)		(1.925)	
200–249	− 0.217	− 0.014	− 1.522	− 0.482	− 1.110***	− 0.165				
	(0.342)		(1.010)		(0.402)					
*Firm age* (base: < 1 year)										
1–2 years	− 0.108	− 0.009	0.484	0.154	0.173	0.048	0.535	0.055	− 1.409**	− 0.081
	(0.139)		(0.453)		(0.173)		(0.459)		(0.692)	
2–5 years	− 0.027	− 0.003	0.558	0.175	− 0.062	− 0.016	0.423	0.046	− 0.137	− 0.003
	(0.141)		(0.438)		(0.174)		(0.524)		(0.735)	
6–9 years	− 0.244*	− 0.019	0.688	0.210	− 0.093	− 0.024	− 0.183	− 0.028	− 0.888	− 0.034
	(0.144)		(0.472)		(0.181)		(0.424)		(0.658)	
10–15 years	− 0.110	− 0.010	0.335	0.109	− 0.243	− 0.058	0.037	0.005	− 1.516**	− 0.094
	(0.138)		(0.428)		(0.173)		(0.466)		(0.686)	
> 15 years	− 0.100	− 0.009	0.660*	0.203	− 0.316*	− 0.074	0.260	0.031	− 1.667**	− 0.115
	(0.131)		(0.400)		(0.162)		(0.447)		(0.649)	
*Legal status* (base: sole proprietorship)										
Partnership	− 0.042	− 0.003	− 0.212	− 0.064	0.122	0.029	− 0.114	− 0.017	0.507	0.025
	(0.077)		(0.233)		(0.170)		(0.535)		(0.443)	
LLP	− 0.259**	− 0.017	0.123	0.034	− 0.002	0.000				
	(0.106)		(0.414)		(0.283)					
LTD Co	− 0.021	− 0.002	0.116	0.032	0.125	0.030	0.441*	0.049	− 0.619*	− 0.057
	(0.066)		(0.187)		(0.098)		(0.236)		(0.363)	
*Industry* (base: agriculture)										
Manufacturing	− 0.112	− 0.010	0.301	0.090	0.046	0.008	− 0.489	− 0.073	− 0.949*	− 0.079
	(0.108)		(0.312)		(0.183)		(0.460)		(0.576)	
Construction	− 0.138	− 0.013	0.527*	0.149	0.336**	0.069	− 0.031	− 0.004	0.305	0.012
	(0.099)		(0.316)		(0.164)		(0.449)		(0.505)	
Wholesale/retail	− 0.044	− 0.004	0.014	0.004	0.203	0.039	0.482	0.041	− 1.171**	− 0.110
	(0.103)		(0.309)		(0.159)		(0.492)		(0.503)	
Hotel and catering	− 0.093	− 0.009	0.409	0.119	0.340**	0.070	0.693	0.052	− 1.662***	− 0.201
	(0.101)		(0.315)		(0.163)		(0.469)		(0.629)	
Trans and Comms	− 0.187*	− 0.016	0.331	0.098	0.672***	0.158	− 0.527	− 0.081	− 0.559	− 0.037
	(0.111)		(0.339)		(0.177)		(0.517)		(0.535)	
Business service	− 0.222**	− 0.019	0.221	0.067	0.425***	0.091	0.050	0.006	− 0.440	− 0.027
	(0.096)		(0.319)		(0.161)		(0.470)		(0.473)	
Health	− 0.326**	− 0.025	0.605	0.167	0.439**	0.094	0.724	0.053	− 0.435	− 0.027
	(0.127)		(0.477)		(0.216)		(0.603)		(0.683)	
Other services	− 0.028	− 0.003	0.139	0.043	0.144	0.027	0.498	0.042	− 0.872*	− 0.069
	(0.106)		(0.310)		(0.170)		(0.436)		(0.512)	
*Region* (base: Scotland)										
North East	− 0.031	− 0.003	0.870**	0.276	− 0.094	− 0.024	0.805	0.049	− 0.787	− 0.195
	(0.134)		(0.381)		(0.215)		(0.527)		(0.691)	
York and Humber	− 0.024	− 0.002	0.543	0.182	− 0.072	− 0.019	− 0.180	− 0.020	1.460**	0.186
	(0.114)		(0.340)		(0.174)		(0.471)		(0.597)	
North West	− 0.077	− 0.006	0.773**	0.250	− 0.052	− 0.014	− 0.305	− 0.037	1.494***	0.188
	(0.115)		(0.346)		(0.184)		(0.557)		(0.498)	
West Midlands	− 0.069	− 0.006	1.030***	0.316	0.032	0.009	− 0.434	− 0.056	1.317***	0.177
	(0.113)		(0.375)		(0.177)		(0.475)		(0.485)	
East Midlands	− 0.039	− 0.003	0.799**	0.257	− 0.093	− 0.024	0.468	0.035	2.418***	0.216
	(0.116)		(0.374)		(0.181)		(0.444)		(0.605)	
East England	− 0.112	− 0.009	0.710**	0.232	− 0.312*	− 0.074	− 0.844*	− 0.136	1.059	0.157
	(0.115)		(0.334)		(0.185)		(0.487)		(0.696)	
Wales	− 0.040	− 0.003	0.486	0.164	− 0.085	− 0.022	− 0.211	− 0.024	0.957*	0.147
	(0.117)		(0.364)		(0.187)		(0.522)		(0.529)	
South West	0.047	0.004	0.706**	0.231	− 0.345*	− 0.081	0.766	0.048	0.510	0.092
	(0.110)		(0.322)		(0.188)		(0.491)		(0.487)	
London	− 0.085	− 0.007	0.631*	0.209	− 0.178	− 0.044	0.002	0.000	1.107***	0.161
	(0.107)		(0.324)		(0.165)		(0.477)		(0.390)	
South East	− 0.031	− 0.003	1.083***	0.329	− 0.203	− 0.050	0.127	0.012	0.772*	0.127
	(0.105)		(0.299)		(0.169)		(0.432)		(0.425)	
N. Ireland	− 0.154	− 0.012	0.411	0.140	− 0.160	− 0.040	− 0.841*	− 0.135	0.659	0.113
	(0.142)		(0.468)		(0.203)		(0.505)		(0.653)	
*PROFIT*	− 0.089	− 0.007	− 0.134	− 0.038	0.054	0.013	− 0.263	− 0.029	1.143***	0.104
	(0.056)		(0.156)		(0.091)		(0.224)		(0.257)	
*Risk rating* (base: minimum)										
Low	0.189*	0.012	− 0.056	− 0.016			0.382	0.059	0.680*	0.054
	(0.097)		(0.312)				(0.369)		(0.372)	
Average	0.217**	0.014	0.046	0.013			0.502	0.074	0.282	0.027
	(0.093)		(0.333)				(0.383)		(0.443)	
Above average	0.293***	0.020	− 0.085	− 0.024			0.741*	0.097	− 0.115	− 0.013
	(0.096)		(0.350)				(0.443)		(0.504)	
Not known	0.226**	0.015	0.076	0.021			0.172	0.029		
	(0.108)		(0.358)				(0.482)			
*Sales growth* (base: > 40%)										
20 to 40%	− 0.191	− 0.022								
	(0.161)									
0 to 20%	− 0.277*	− 0.030								
	(0.156)									
No growth	− 0.426***	− 0.041								
	(0.155)									
< 0%	− 0.175	− 0.020								
	(0.157)									
*FASTGROW*	0.078	0.006								
	(0.132)									
*Banking relationship* (base: strong)										
Fair	0.158**	0.010	0.436**	0.148						
	(0.065)		(0.205)							
Weak	0.595***	0.056	1.111***	0.319						
	(0.077)		(0.224)							
*AIMGROW*					0.170**	0.041				
					(0.081)					
*FINPROBLEM*					0.415***	0.099				
					(0.098)					
Inverse Mills ratios										
*Historical rejection*			− 0.065	− 0.018	− 1.244***	− 0.296	1.007	0.109	− 1.607	− 0.147
			(0.695)		(0.368)		(0.944)		(1.035)	
* SOUGHT*							0.998*	0.109	− 0.291	− 0.026
							(0.536)		(0.723)	
* GOT*									− 1.458	− 0.133
									(1.148)	
Constant	− 1.371***		− 1.337		0.239		− 4.326*		7.945**	
	(0.261)		(1.703)		(0.758)		(2.300)		(3.556)	
N Obs	30,577		1077		30,577		30,577		30,577	
Censored N					5,052		1026		867	
Wald *χ*^2^	137.18***		158.86***		260.90***		125.54***		114.59***	
Pseudo *R*^2^	0.026		0.147		0.120		0.250		0.364	
Log likelihood	− 555.75		− 67.16		− 253.54		− 18.45		− 12.27	

Models 1 and 2 estimate historical rejection and scarring prior to Covid-19. Models 3 to 5 estimate loan demand, loan supply and use of government-guaranteed loan schemes during Covid-19. Full regression results including the selection equations are reported in the Online Appendix. * p < 0.10; ** *p* < 0.05; *** *p* < 0.01. Asymptotic robust standard errors reported. Weights applied

### Previous loan rejection

We initially found that only a very small subset of small firms, around 1.18% of the total business population and 8.4% of the borrower population, had a previous negative loan application outcome. But of the rejected borrower population, 72.4% were scarred and had become reluctant to make future loan applications. Given that the general loan refusal rate in stable economic times is around 10% (Krasniqi, 2010; Scott, 2006) and in this sample 8.4%, this implies that a substantial share of the rejected set of firms had received more than one loan rejection in the past. This strengthens the potential effect of borrower scarring, which is central to our study.

The results (Model 1) show that micro businesses (1–9 employees) have a higher probability of having a historical loan rejection, by 10%, compared to all other size classes of firm. This is consistent with information-based problems for the smallest of firms. In contrast, real estate businesses have a lower rejection probability than firms in all other industry sectors. Risk rating plays a key role in determining loan rejection. Compared to firms with minimum risk, all other firms have a significantly lower probability of having their loan application accepted, by magnitudes between 12 and 20%. Rejection probability is highest amongst firms with above average risk. This is consistent with previous empirical work on loan rejection (Sannajust, 2014) and with standard bank lending processes which place credit risk at the forefront of their decision-making process’s (Berg, 2018). We also note that firms with low or no sales growth were less likely to experience historical rejection, implying that banks care more about the volatility of growth, as a proxy for business risk, than the performance of a single year when making lending decisions. Lastly, a good relationship with the main bank significantly reduces the odds of loan rejection. Compared to those with strong banking relationship, the chance of historical rejection is increased by 5.6 percentage points, or more than doubled (mean rejection = 3.7% from Table 1), for firms with a weak banking relationship. This suggests that building a strong relationship with a main bank creates the conditions for a mutually beneficial relationship where softer information can be shared.

### Scarring from previous loan rejection

Here, we distinguish (Model 2) between firms that were rejected for loans in the pre-Covid-19 period and then became scarred in terms of reporting a reluctance to make any future loan applications which we term *Scarred Borrowers*. We use Model 1 as the selection equation to calculate the IMR. Two measures of historical performance, sales growth and *FASTGROW*, are used as the exclusion restrictions. Here, we contend that the intention of finance seeking is more associated with the firm’s *subjective* perception of credit supply, but lenders would use more *objective* measures of performance and riskiness to make lending decisions.

The first point of note is that there is no evidence of a selection effect, since the IMR is statistically insignificant. From the results, it is very clear that the probability of scarring diminishes increasingly in firm size class. Thus, once a micro business has received a loan application rejection, it is much more likely never to make an application again in the future. Scarring is also higher amongst the oldest age class of businesses (> 15 years since start) which suggests that start-up and early-stage firms are less prone to scarring through loan rejection.

There is evidence that the construction sector had a higher probability of scarring although the reasons for this industry effect are unclear. However, there was tremendous variation in the probability of scarring across the regions of the UK with each single region having a significant and different coefficient. On this, firms located in the South East of England (the most prosperous region of the UK) had the single highest probability of being scarred by a loan rejection. This may relate to expectations as firms in poor regions might expect rejection and cope with it better. In general, there is no clear pattern across UK regions based on relative economic prosperity which reinforces our general argument that behaviours and changing behaviours are a combination of many factors including a spatial one.

### Current Covid-19 loan demand

Model 3 estimates the probability that a firm demanded a loan during the first two quarters of the Covid-19 crisis (2020 q2 and q3) and whether such demand differs for SMEs scarred by previous rejection. We use the same selection adjustment as in Model 2, and the significantly negative coefficient estimate of the IMR justifies the treatment of sample selection. This is also consistent with the previous empirical evidence that historical rejection would discourage loan application.

The key finding is that scarring due to historical rejection is positively associated with loan application during the Covid-19 crisis. Scarred borrowers were 16% more likely to apply, an 80% increase compared to the average loan application probability of 19%. In this sense, the severity of the crisis in terms of its impact on firms through reduced trading and the associated income losses and cash flow pressures have obviously changed the behaviours of firms that were previously scarred by rejection. Financing constraints have become more severe for scarred borrowers, and cash flow pressure accumulated through historical non-borrowing means they can no longer rely on internal finance to fund the business. With liquidity problems exacerbated as businesses faced restricted trading conditions and lower revenues in the formal lockdown further during the pandemic, the financing shortfall soon extended to the whole small business sector: the demand for bank loans increased by 6% during Q3 2020 and thus H1 is supported. If this key behaviour was sustained into the post-Covid-19 period, then we would expect to see an upward shift in the general demand for bank loans which had reached a historical low in the period from the Brexit vote in June 2016 through to the onset of Covid-19 in March 2020. Therefore, we find strong support for H3.

The notable feature of these results was an inverted ‘U’-shaped pattern in loan demand as firm size increases. Medium- (100–199 employees) and large–medium (200–249 employees) firms are on average 15% less likely to seek external loans than their smaller counterparts. This is consistent with earlier UK empirical evidence which identified a precautionary savings gap amongst these size classes of business which meant that they were in the most precarious liquidity position entering the Covid-19 crisis (Cowling, Brown and Rocha, 2002; Brown & Cowling, 2021). The evidence suggests that this weakened cash flow position led to an increase in the demand for loans during the Covid-19 crisis. There is also a clear sector effect on within-pandemic loan demand. Businesses experiencing more severe disruptions because of the lockdown, such as construction, hotel and catering and transportation and communications, exhibited higher demand caused by the significant loss of revenue during the pandemic. The higher application rate for the health industry is likely to be a result of inflated demand for health-related products and services in the Covid-19 crisis.

### Loan supply and use of government-guaranteed schemes

Model 4 reports the coefficient estimates of the loan acceptance equation. As discussed previously, the selection process includes two equations, historical rejection which is the same as before and loan application. For the second selection equation, entrepreneurial growth objectives (*AIMGROW*) and the perception of cash flow problems as the main growth obstacle (*FINPROBLEM*) are used as exclusion restrictions. Growth objectives are commonly used as the exclusion restriction in previous credit rationing studies (c.f. Cowling et al., 2016), as they are obviously unobservable by lenders but found to be significant in explaining the finance-seeking behaviour by individual businesses (Michaelas et al., 1999; Psillaki and Daskalakis, 2009). The use of the second exclusion restriction is justified by the relationship between cash constraints and external finance need according to the pecking order theory (Myers & Majluf, 1984).

Both exclusion restrictions are positively and significantly associated with loan application (Table A2), but the coefficient estimates of the two IMRs show little evidence of sample selection bias. The marginally significant and positive IMR for the loan application equation suggests that finance demand was generally based on an unbiased estimation of the true loan approval probability. Our headline finding is that applicants scarred by previous rejection were significantly less likely to be approved, by a margin of 14%. This translates to 7,300 more rejections^3^ out of 6 million SME population compared to businesses not scarred by previous rejection. This suggests that whilst scarred SMEs were forced to seek external finance during the pandemic due to more severe financial constraints, they appeared to be more disadvantaged as lenders treated their previous rejection as a negative signal of borrower quality.

There is no evidence of changes in conventional loan supply during the pandemic, and thus H2 is not supported. We have noted that the overwhelming majority of business lending offers in the Covid-19 crisis were backed by the government guarantee schemes. In this sense, despite general model significance, it was difficult to distinguish between firms who got a loan offer and those who got refused by section, region or credit risk. Banks are shown to have relied on a few common indicators, primarily sales and business size, when processing loan applications. Firms with higher sales were more likely to be approved, implying that application decisions were generally based on an unbiased evaluation of the true approval probability. On the other hand, loan rejection increased monotonically with the number of employees. This suggests that during the pandemic, banks have imposed stronger restrictions in lending to larger firms which normally have higher capital requirements.

Model 5 examines government-guaranteed lending. One additional mechanism enters the selection process because loan type is only observed if a loan application is approved. We use firm-bank relationship to identify the selection equation, drawn from the extant relationship lending literature on commercial bank lending (Berger & Udell, 1995 and 2002). Similarly, although the exclusion restrictions seem to be significant (Table A2), all three IMRs are insignificant, showing no evidence of selection bias.

We find that the offer of government guarantee was not discriminating against scarred borrowers, and there is no sign of more intense government support the deeper into the crisis; thus, H4 is also not supported. However, there was a clearer differentiation with respect to the targets of public support. Micro (1–9 employees) and start-up (< 1 year) businesses had a higher, whilst limited liability companies had a lower likelihood to receive government-guaranteed loans. There was also evidence of businesses located in more deprived regions (York and Humberside, North West, West and East Midlands) having a higher probability of receiving a government-guaranteed loan offer. Surprisingly, sectors worst hit by the shutdown of businesses during the pandemic, namely wholesale/retail and hotel/catering, were the least likely to receive government support. This may relate to the wage protection schemes available which covered 80% of pay for workers in sectors forced to lock down and the relatively low capital requirements in service sectors. Regarding credit risk, we find that the probability of receiving a government-guaranteed loan offer was higher for low-risk firms and then decreasing for average- and above average-risk firms. This implies that the government only had a moderate risk tolerance, and the recoverability of public funds was a key criterion for government supporting initiatives. It also suggests that banks still conducted due diligence on loan applications.

Given the low rejection rate on loan application and the low percentage of commercial loans approved (Fig. 2), the legitimacy of conventional binary (probit or logit) regressions may be compromised by the ‘rare-event’ problem first noted by King and Zeng (2001). To test the robustness of our results against potential small sample bias, we re-estimate Models 4 and 5 in Table 2 using the penalised maximum likelihood estimation proposed by Firth (1993), as statistical simulations have found this method of bias correction to be more effective with large sample size (Leitgöb, 2013). As reported in Table A3, our headline findings regarding the effect of borrower scarring on general and government-backed loan approval and the distribution of government lending with respect to various business characteristics still hold.

## Conclusion

We set out to establish how important a previous loan rejection and any scarring that might arise from a rejection was in informing the small firms’ decision on whether to make a new loan application during the Covid-19 crisis. We then traced out the process from application for a loan to the banks’ decision and potential offer of a government-backed Covid-19-guaranteed lending scheme. We find that over the last 10 years, the loan rejection rate was fairly constant even though there was variation in loan demand. But for those businesses that had a rejection, there was evidence of scarring in the sense that they changed their expectations about the probability that future loan applications would be accepted to such an extent that they became reluctant to even apply. This subset of the business population was estimated to be 6.51% of the potential borrower pool and 77.5% of rejected borrowers. They were found to be highly concentrated amongst micro and small businesses and businesses that had gone beyond their formative stages. This suggests that they were shocked by a rejection given their relatively advanced stage in their life cycle. At this stage, their expectations based on track record would have been high regarding a positive outcome.

We then explored their borrowing behaviours during the Covid-19 crisis when the vast majority of firms faced reduced trading activity, significant income reductions and problems with cash `flow. Prior research found that micro and small businesses were disproportionately impacted on all three measures and we established that it was precisely these size classes of firm that had the highest loan demand in the first two quarters of the Covid-19 crisis which began in March 2020 in the UK. Importantly, we found no evidence that prior rejection OR scarring from prior rejection impacted on the desire to make a Covid-19 period loan application. In this sense, the scarring effect which influenced borrowing behaviours dissipated when firms were faced with the sheer magnitude of the economic shock wrought by Covid-19.

On the supply side of the loan market, banks were more than willing to make loan offers to firms that had experienced historical loan rejections suggesting that they treat each funding proposal, even from the same firm, on its merits. However, the presence of the two most generous government-backed guaranteed loan schemes (CBILS and BBLS) may have given banks the leeway to drop their lending standards when in a normal crisis they would have raised them and choked off lending. In terms of routing loan applications through normal lending channels or government-guaranteed loan schemes, again, we find no evidence that formerly rejected or scarred borrowers were routed onto government-backed lending schemes.

In terms of what these findings might mean for a future post-Covid-19 world, we tentatively suggest that the small, but non-trivial, group of scarred borrowers may have revised their expectations about banks and lending upward. If this was the case, then we would see a structural shift in the potential borrower pool with a greater share of the business population willing to make bank loan applications. It remains the case that when the government guarantee schemes cease to exist, if banks raise their lending standards and begin to choke off lending, then we could potentially be back to square one. In parallel with this de-scarring, we also observed a decline in the share of what have been termed permanent non-borrowers. Again, if this group of businesses sustained their new found willingness to apply for loans, then the pool of borrowers would expand significantly. However, given the strong evidence of a financial pecking order where internal funds are preferred over all external debt (and equity particularly), any expansion in the economy which puts more cash into the small business sector would see them return to their self-financing status quo.

In terms of our contribution, we think we have added a new and important dimension to the wider debates and research around credit rationing by broadening the focus of the issue to consider how previous credit rationing then shapes future behaviours and effectively increases pool of small firms operating on the first step of the pecking order ladder which is financing all operations and investment from internal resources. We think that previous literature has identified borrower discouragement as an important phenomenon, but the concept of the scarred borrower adds nuance and additional insights. Indeed, some previously rejected loan applicants become scarred and some do not which is interesting in its own right. Furthermore, credit rationing will always be more strongly associated with smaller (and younger) firms due to their information opacity.

The simple UK Covid-19 loan guarantee take-up figures show that 971,302 small firms accessed loans on the Bounce Back Scheme compared to 76,704 firms for the mid-range scheme (CBILS), and 569 large firms on the big firm scheme (CLBILS) reinforce this point. In the wider context of firm size, small firms when seeking external finance are heavily reliant on debt. Medium and large firms increasingly have much greater access to the full range of debt and equity sources which means they are less dependent upon a single source. They also have far higher approval rates for all sources of finance. So, although the UK does have a well-developed loan market, it has always been the case that its transactional lending and banking structure means that the ability of a firm to provide collateral against loans is central to the banks’ decision. Therefore, we have had a loan guarantee scheme for small firms since 1981 and current discussions with UK policymakers have stressed the importance of maintaining a public loan guarantee scheme in the post-Covid-19 period and enhancing its capacity in a crisis period.

There are also clear implications for small businesses themselves. First, developing and maintaining a strong relationship with ones’ main bank is important for reducing information asymmetries and increasing the probability of future loan application success. Furthermore, banks treat each loan application in isolation at a specific point in time and their decision reflects the prevailing economic conditions at that time, although a poor application will be treated as such at any time. So, some previous loan rejections may simply mean that economic conditions were poor or that the funding proposal was deemed inadequate. Returning later with a new funding proposal will be treated on its relative merits at that time; thus, we encourage entrepreneurs not to simply drop out of the loan market as that may damage their firm by reducing potentially profitable new investment.

## Supplementary Information

Below is the link to the electronic supplementary material.

Supplementary file1 (DOCX 65 KB)

## Footnotes

1 The missing values are a result of including additional controls, and primarily come from the sales data. A third of the responses on sales are ‘don’t know’ or ‘refused’, and were subsequently treated as missing values. We confirm the robustness of our results with respect to the missing data, by treating observations with missing sales data as a separate category (results available upon request).

2 To save space, Table 2 only reports the coefficient estimates of the outcome equations when sample selection is considered. The full regression results including the selection equations are reported in the Online Appendix Table A2.

3 Fourteen percent (Table 2) of 0.85% (Fig. 1) SME population.
