# Supplementary material for: Has previous loan rejection scarred firms from applying for loans during Covid-19?
Source: Small Bus Econ (Dordr). 2021 Dec 18;59(4):1327–50. doi: 10.1007/s11187-021-00586-2 (PMC8683813; doi:10.1007/s11187-021-00586-2)
Supplement: Supplementary file 1 — Supplementary file1 (DOCX 65 KB) [file 11187_2021_586_MOESM1_ESM.docx]

**Online Appendix**

Table A1: Loan demand, supply and scarring by region and sector (Weighted)

|  | **Panel A: Regional Differences in Access to Finance Measures** | | | | | | | | | | | | | | | | | | | | | | | |
| --- | --- | --- | --- | --- | --- | --- | --- | --- | --- | --- | --- | --- | --- | --- | --- | --- | --- | --- | --- | --- | --- | --- | --- | --- |
|  | Scotland | | North/North East | | York/Humberside | | North West | | West Midlands | | East Midlands | | East of Engand | | Wales | | South West | | London | | South East | | Northern Ireland | |
|  | Mean | s.d. | Mean | s.d. | Mean | s.d. | Mean | s.d. | Mean | s.d. | Mean | s.d. | Mean | s.d. | Mean | s.d. | Mean | s.d. | Mean | s.d. | Mean | s.d. | Mean | s.d. |
| **Dependent Variables** |  |  |  |  |  |  |  |  |  |  |  |  |  |  |  |  |  |  |  |  |  |  |  |  |
| *SOUGHT* | 0.206 | 0.405 | 0.158 | 0.365 | 0.198 | 0.399 | 0.176 | 0.381 | 0.204 | 0.404 | 0.189 | 0.392 | 0.110 | 0.313 | 0.174 | 0.380 | 0.131 | 0.337 | 0.167 | 0.373 | 0.138 | 0.345 | 0.159 | 0.366 |
| *GOT* | 0.921 | 0.272 | 0.967 | 0.180 | 0.897 | 0.305 | 0.915 | 0.280 | 0.913 | 0.284 | 0.947 | 0.226 | 0.799 | 0.403 | 0.922 | 0.271 | 0.977 | 0.152 | 0.941 | 0.236 | 0.969 | 0.173 | 0.823 | 0.385 |
| *GOT_GOVT* | 0.817 | 0.389 | 0.806 | 0.401 | 0.966 | 0.182 | 0.958 | 0.202 | 0.916 | 0.279 | 0.998 | 0.040 | 0.879 | 0.328 | 0.813 | 0.393 | 0.967 | 0.181 | 0.893 | 0.310 | 0.958 | 0.202 | 0.818 | 0.390 |
| Historical Rejection | 0.038 | 0.192 | 0.031 | 0.173 | 0.030 | 0.171 | 0.031 | 0.174 | 0.032 | 0.175 | 0.030 | 0.172 | 0.026 | 0.158 | 0.032 | 0.175 | 0.038 | 0.192 | 0.030 | 0.170 | 0.033 | 0.178 | 0.024 | 0.154 |
| Scarred by Rejection | 0.525 | 0.502 | 0.761 | 0.431 | 0.757 | 0.431 | 0.752 | 0.434 | 0.817 | 0.389 | 0.760 | 0.429 | 0.719 | 0.452 | 0.677 | 0.470 | 0.724 | 0.449 | 0.713 | 0.454 | 0.850 | 0.359 | 0.680 | 0.471 |
|  |  |  |  |  |  |  |  |  |  |  |  |  |  |  |  |  |  |  |  |  |  |  |  |  |
|  |  |  |  |  |  |  |  |  |  |  |  |  |  |  |  |  |  |  |  |  |  |  |  |  |
|  | **Panel B: Sector Differences in Access to Finance Measures** | | | | | | | | | | | | | | | | | |  |  |  |  |  |  |
|  | Agriculture | | Manufacturing | | Construction | | Wholesale/retail | | Hotel & catering | | Trans & Comms | | Business Service | | Health | | Other services | |  |  |  |  |  |  |
|  | Mean | s.d. | Mean | s.d. | Mean | s.d. | Mean | s.d. | Mean | s.d. | Mean | s.d. | Mean | s.d. | Mean | s.d. | Mean | s.d. |  |  |  |  |  |  |
| *SOUGHT* | 0.150 | 0.357 | 0.152 | 0.359 | 0.162 | 0.369 | 0.222 | 0.416 | 0.240 | 0.427 | 0.232 | 0.422 | 0.160 | 0.367 | 0.123 | 0.329 | 0.150 | 0.357 |  |  |  |  |  |  |
| *GOT* | 0.960 | 0.197 | 0.847 | 0.361 | 0.916 | 0.278 | 0.901 | 0.300 | 0.951 | 0.217 | 0.871 | 0.336 | 0.935 | 0.248 | 0.966 | 0.181 | 0.942 | 0.235 |  |  |  |  |  |  |
| *GOT_GOVT* | 0.883 | 0.324 | 0.899 | 0.303 | 0.967 | 0.178 | 0.935 | 0.247 | 0.942 | 0.235 | 0.906 | 0.293 | 0.930 | 0.256 | 0.987 | 0.113 | 0.910 | 0.287 |  |  |  |  |  |  |
| Historical Rejection | 0.036 | 0.187 | 0.035 | 0.184 | 0.031 | 0.173 | 0.041 | 0.199 | 0.041 | 0.199 | 0.031 | 0.174 | 0.026 | 0.158 | 0.020 | 0.138 | 0.039 | 0.194 |  |  |  |  |  |  |
| Scarred by Rejection | 0.632 | 0.485 | 0.746 | 0.437 | 0.816 | 0.388 | 0.665 | 0.474 | 0.652 | 0.478 | 0.788 | 0.410 | 0.738 | 0.441 | 0.850 | 0.359 | 0.696 | 0.462 |  |  |  |  |  |  |

Table A2: Full Regression Results for Models 4 & 5, Table 2

|  | **(I) Covid-19 Loan Acceptance (*GOT*)** | | | **(II) Covid-19 Government Guarantee (*GOT_GOV*)** | | | |
| --- | --- | --- | --- | --- | --- | --- | --- |
|  | Selection Equations | | Outcome Eq. | Selection Equations | | | Outcome Eq. |
|  | (1) Rejection | (2) SOUGHT | Covid-19 Loan Acceptance | (1) Rejection | (2) SOUGHT | (3) GOT | Covid-19 Gov Guarantee |
| Variables | Coeff. | Coeff. | Coeff. | Coeff. | Coeff. | Coeff. | Coeff. |
| *Scarred by Rejection* |  |  | -1.317*** |  |  |  | -0.522 |
|  |  |  | (0.249) |  |  |  | (0.454) |
| ***Survey Wave*** (Base: Q2 2018) | |  |  |  |  |  |  |
| Q3 2018 | 0.023 |  |  | 0.023 |  |  |  |
|  | (0.104) |  |  | (0.104) |  |  |  |
| Q4 2018 | 0.088 |  |  | 0.088 |  |  |  |
|  | (0.111) |  |  | (0.111) |  |  |  |
| Q1 2019 | -0.160 |  |  | -0.159 |  |  |  |
|  | (0.115) |  |  | (0.115) |  |  |  |
| Q2 2019 | -0.035 |  |  | -0.035 |  |  |  |
|  | (0.106) |  |  | (0.106) |  |  |  |
| Q3 2019 | -0.059 |  |  | -0.059 |  |  |  |
|  | (0.113) |  |  | (0.113) |  |  |  |
| Q4 2019 | -0.168 |  |  | -0.168 |  |  |  |
|  | (0.109) |  |  | (0.109) |  |  |  |
| Q1 2020 | -0.086 |  |  | -0.087 |  |  |  |
|  | (0.110) |  |  | (0.110) |  |  |  |
| Q2 2020 | 0.052 |  |  | 0.052 |  |  |  |
|  | (0.106) |  |  | (0.106) |  |  |  |
| Q3 2020 | -0.018 | 0.217*** | 0.357 | -0.018 | 0.214*** | 0.330** | 0.293 |
|  | (0.108) | (0.078) | (0.231) | (0.109) | (0.077) | (0.157) | (0.303) |
| ln(s*ales*) | 0.008 | 0.258*** | 0.409*** | 0.008 | 0.258*** | 0.290*** | -0.353 |
|  | -0.022 | -0.036 | (0.124) | (0.022) | (0.036) | (0.068) | (0.276) |
| ***Employment Size*** (Base: 1 employee) | |  |  |  |  |  |  |
| 1-9 | 0.119** | -0.036 | -0.538** | 0.120** | -0.035 | -0.543*** | 0.793* |
|  | -0.056 | -0.091 | (0.249) | (0.056) | (0.091) | (0.171) | (0.469) |
| 10-49 | 0.003 | -0.268* | -1.145*** | 0.004 | -0.264* | -0.891*** | 1.049 |
|  | -0.087 | -0.139 | (0.335) | (0.087) | (0.139) | (0.265) | (0.737) |
| 50-99 | -0.174 | -0.621*** | -1.824*** | -0.169 | -0.617*** | -1.246*** | 1.956* |
|  | -0.122 | -0.18 | (0.494) | (0.122) | (0.180) | (0.364) | (1.058) |
| 100-199 | -0.205 | -1.132*** | -3.400*** | -0.205 | -1.128*** | -2.505*** | 3.125 |
|  | -0.155 | -0.246 | (0.769) | (0.155) | (0.246) | (0.521) | (1.925) |
| 200 – 249 | -0.212 | -1.330*** |  | -0.210 | -1.330*** |  |  |
|  | -0.343 | -0.383 |  | (0.343) | (0.383) |  |  |
| ***Firm Age*** (Base: < 1 year) | |  |  |  |  |  |  |
| 1-2 years | -0.11 | 0.049 | 0.535 | -0.113 | 0.057 | 0.433 | -1.409** |
|  | -0.139 | -0.167 | (0.459) | (0.139) | (0.166) | (0.345) | (0.692) |
| 2-5 years | -0.026 | 0.126 | 0.423 | -0.027 | 0.133 | 0.136 | -0.137 |
|  | -0.141 | -0.167 | (0.524) | (0.140) | (0.167) | (0.347) | (0.735) |
| 6-9 years | -0.244* | -0.187 | -0.183 | -0.248* | -0.180 | -0.095 | -0.888 |
|  | -0.144 | -0.179 | (0.424) | (0.144) | (0.178) | (0.345) | (0.658) |
| 10-15 years | -0.108 | -0.2 | 0.037 | -0.109 | -0.192 | -0.155 | -1.516** |
|  | -0.138 | -0.172 | (0.466) | (0.138) | (0.172) | (0.360) | (0.686) |
| >15 years | -0.099 | -0.292* | 0.260 | -0.098 | -0.284* | 0.052 | -1.667** |
|  | -0.131 | -0.161 | (0.447) | (0.130) | (0.161) | (0.348) | (0.649) |
| ***Legal Status*** (Base: sole proprietorship) | | |  |  |  |  |  |
| Partnership | -0.042 | 0.05 | -0.114 | -0.042 | 0.052 | 0.030 | 0.507 |
|  | -0.077 | -0.164 | (0.535) | (0.077) | (0.163) | (0.375) | (0.443) |
| LLP | -0.259** | -0.33 |  | -0.259** | -0.329 |  |  |
|  | -0.106 | -0.268 |  | (0.106) | (0.268) |  |  |
| LTD Co. | -0.021 | 0.064 | 0.441* | -0.022 | 0.066 | 0.405** | -0.619* |
|  | -0.066 | -0.097 | (0.236) | (0.066) | (0.097) | (0.180) | (0.363) |
| ***Industry*** (Base: Agriculture) | |  |  |  |  |  |  |
| Manufacturing | -0.112 | -0.001 | -0.489 | -0.114 | -0.002 | -0.425 | -0.949* |
|  | -0.108 | -0.18 | (0.460) | (0.108) | (0.179) | (0.361) | (0.576) |
| Construction | -0.139 | 0.257 | -0.031 | -0.139 | 0.257 | -0.005 | 0.305 |
|  | -0.099 | -0.162 | (0.449) | (0.099) | (0.161) | (0.328) | (0.505) |
| Wholesale/retail | -0.042 | 0.246 | 0.482 | -0.041 | 0.245 | 0.223 | -1.171** |
|  | -0.103 | -0.159 | (0.492) | (0.103) | (0.158) | (0.356) | (0.503) |
| Hotel & catering | -0.094 | 0.364** | 0.693 | -0.095 | 0.369** | 0.483 | -1.662*** |
|  | -0.101 | -0.163 | (0.469) | (0.101) | (0.162) | (0.366) | (0.629) |
| Trans & Comms | -0.186* | 0.563*** | -0.527 | -0.184* | 0.566*** | -0.311 | -0.559 |
|  | -0.111 | -0.172 | (0.517) | (0.111) | (0.172) | (0.357) | (0.535) |
| Business Service | -0.225** | 0.259* | 0.050 | -0.225** | 0.258* | 0.193 | -0.440 |
|  | -0.096 | -0.153 | (0.470) | (0.096) | (0.153) | (0.308) | (0.473) |
| Health | -0.324** | 0.144 | 0.724 | -0.323** | 0.146 | 0.612 | -0.435 |
|  | -0.127 | -0.192 | (0.603) | (0.127) | (0.191) | (0.410) | (0.683) |
| Other services | -0.027 | 0.191 | 0.498 | -0.026 | 0.190 | 0.203 | -0.872* |
|  | -0.106 | -0.168 | (0.436) | (0.106) | (0.168) | (0.335) | (0.512) |
| ***Region*** (Base: Scotland) |  |  |  |  |  |  |  |
| North East | -0.026 | -0.112 | 0.805 | -0.027 | -0.112 | 0.361 | -0.787 |
|  | -0.134 | -0.211 | (0.527) | (0.134) | (0.211) | (0.399) | (0.691) |
| York & Humber | -0.018 | -0.074 | -0.180 | -0.019 | -0.079 | -0.203 | 1.460** |
|  | -0.114 | -0.172 | (0.471) | (0.114) | (0.172) | (0.361) | (0.597) |
| North West | -0.074 | -0.087 | -0.305 | -0.075 | -0.085 | -0.301 | 1.494*** |
|  | -0.115 | -0.18 | (0.557) | (0.115) | (0.179) | (0.398) | (0.498) |
| West Midlands | -0.063 | -0.009 | -0.434 | -0.066 | -0.004 | -0.347 | 1.317*** |
|  | -0.112 | -0.172 | (0.475) | (0.112) | (0.172) | (0.357) | (0.485) |
| East Midlands | -0.037 | -0.134 | 0.468 | -0.039 | -0.136 | 0.284 | 2.418*** |
|  | -0.116 | -0.177 | (0.444) | (0.116) | (0.177) | (0.334) | (0.605) |
| East England | -0.112 | -0.397** | -0.844* | -0.118 | -0.395** | -0.698* | 1.059 |
|  | -0.114 | -0.179 | (0.487) | (0.114) | (0.178) | (0.363) | (0.696) |
| Wales | -0.04 | -0.111 | -0.211 | -0.041 | -0.110 | -0.291 | 0.957* |
|  | -0.117 | -0.186 | (0.522) | (0.117) | (0.185) | (0.401) | (0.529) |
| South West | 0.048 | -0.248 | 0.766 | 0.047 | -0.249 | 0.323 | 0.510 |
|  | -0.109 | -0.181 | (0.491) | (0.109) | (0.181) | (0.371) | (0.487) |
| London | -0.084 | -0.247 | 0.002 | -0.084 | -0.249 | -0.085 | 1.107*** |
|  | -0.106 | -0.16 | (0.477) | (0.106) | (0.160) | (0.350) | (0.390) |
| South East | -0.026 | -0.196 | 0.127 | -0.027 | -0.198 | 0.067 | 0.772* |
|  | -0.105 | -0.167 | (0.432) | (0.105) | (0.167) | (0.335) | (0.425) |
| N.Ireland | -0.157 | -0.316 | -0.841* | -0.159 | -0.310 | -0.472 | 0.659 |
|  | -0.142 | -0.194 | (0.505) | (0.143) | (0.194) | (0.429) | (0.653) |
| *PROFIT* | -0.09 | -0.069 | -0.263 | -0.089 | -0.068 | -0.089 | 1.143*** |
|  | -0.056 | -0.083 | (0.224) | (0.056) | (0.083) | (0.145) | (0.257) |
| ***Risk Rating*** (Base: Minimum) | |  |  |  |  |  |  |
| Low | 0.187* |  | 0.382 | 0.185* |  | 0.029 | 0.680* |
|  | -0.096 |  | (0.369) | (0.096) |  | (0.274) | (0.372) |
| Average | 0.217** |  | 0.502 | 0.217** |  | 0.168 | 0.282 |
|  | (0.092) |  | (0.383) | (0.093) |  | (0.255) | (0.443) |
| Above Average | 0.290*** |  | 0.741* | 0.291*** |  | 0.276 | -0.115 |
|  | (0.095) |  | (0.443) | (0.096) |  | (0.273) | (0.504) |
| Not Known | 0.225** |  | 0.172 | 0.226** |  | -0.065 |  |
|  | (0.107) |  | (0.482) | (0.107) |  | (0.308) |  |
| ***Sales Growth*** (Base: >40%) | |  |  |  |  |  |  |
| 20% to 40% | -0.196 |  |  | -0.195 |  |  |  |
|  | (0.161) |  |  | (0.161) |  |  |  |
| 0 to 20% | -0.283* |  |  | -0.285* |  |  |  |
|  | (0.155) |  |  | (0.155) |  |  |  |
| No growth | -0.432*** |  |  | -0.433*** |  |  |  |
|  | (0.154) |  |  | (0.154) |  |  |  |
| < 0% | -0.186 |  |  | -0.191 |  |  |  |
|  | (0.157) |  |  | (0.156) |  |  |  |
| *FASTGROW* | 0.067 |  |  | 0.066 |  |  |  |
|  | (0.132) |  |  | (0.131) |  |  |  |
| ***Banking Relationship*** (Base: Strong) | |  |  |  |  |  |  |
| Fair |  |  |  |  |  | -0.366** |  |
|  |  |  |  |  |  | (0.155) |  |
| Weak |  |  |  |  |  | -0.196 |  |
|  |  |  |  |  |  | (0.206) |  |
| *AIMGROW* |  | 0.194** |  |  | 0.197** |  |  |
|  |  | (0.081) |  |  | (0.077) |  |  |
| *FINPROBLEM* |  | 0.419*** |  |  | 0.418*** |  |  |
|  |  | (0.098) |  |  | (0.095) |  |  |
| **Inverse Mills Ratios** |  |  |  |  |  |  |  |
| Historical Rejection |  |  | 1.007 |  |  |  | -1.607 |
|  |  |  | (0.944) |  |  |  | (1.035) |
| *SOUGHT* |  |  | 0.998* |  |  |  | -0.291 |
|  |  |  | (0.536) |  |  |  | (0.723) |
| *GOT* |  |  |  |  |  |  | -1.458 |
|  |  |  |  |  |  |  | (1.148) |
| **Constant** | -1.357*** | -1.983*** | -4.326* | -1.354*** | -1.992*** | -0.706 | 7.945** |
|  | (0.259) | (0.259) | (2.300) | (0.259) | (0.258) | (0.739) | (3.556) |
| N Obs |  |  | 30,577 |  |  |  | 30,577 |
| Censored N |  |  | 1026 |  |  |  | 867 |
| Wald *χ*2 |  |  | 125.54*** |  |  |  | 114.59*** |
| Pseudo R2 |  |  | 0.250 |  |  |  | 0.364 |
| Log likelihood |  |  | -18.45 |  |  |  | -12.27 |
| *ρ*_12_ |  |  | 0.282*** |  |  |  | 0.282*** |
| *ρ*_13_ |  |  |  |  |  |  | -0.280* |
| *ρ*_23_ |  |  |  |  |  |  | 0.842*** |

** p <* .10; ** *p* < .05; *** *p* < .01. Asymptotic robust standard errors reported. Weights applied.

table A3: Rare-Event Logit Regression Using the Firth (2003) Model

|  | **(1) Offered Finance** | | **(2) Offered Government Finance** | |
| --- | --- | --- | --- | --- |
| Variables | Coeff. | dy/dx | Coeff. | dy/dx |
| ***Scarred by Rejection*** | -1.600*** | -0.120 | -0.274 | -0.020 |
|  | (0.323) |  | (0.587) |  |
| ***Survey Wave*** |  |  |  |  |
| Q3 2020 | 0.754** | 0.063 | 0.439 | 0.034 |
|  | (0.306) |  | (0.383) |  |
| ln(s*ales*) | 0.649*** | 0.049 | -0.095 | -0.007 |
|  | (0.194) |  | (0.297) |  |
| ***Employment Size*** (Base: 1 employee) |  |  |  |  |
| 1-9 | -0.271 | -0.015 | 1.003 | 0.084 |
|  | (0.434) |  | (0.649) |  |
| 10-49 | -0.747 | -0.050 | 0.665 | 0.062 |
|  | (0.553) |  | (0.854) |  |
| 50-99 | -1.916** | -0.187 | 0.620 | 0.059 |
|  | (0.799) |  | (1.162) |  |
| 100-199 | -4.194*** | -0.611 | 0.743 | 0.068 |
|  | (1.037) |  | (2.196) |  |
| ***Firm Age*** (Base: < 1 year) |  |  |  |  |
| 1-2 years | 0.864 | 0.060 | -0.554 | -0.043 |
|  | (0.713) |  | (1.021) |  |
| 2-5 years | 1.468** | 0.084 | 0.805 | 0.036 |
|  | (0.728) |  | (1.127) |  |
| 6-9 years | 0.032 | 0.003 | -0.304 | -0.021 |
|  | (0.666) |  | (1.000) |  |
| 10-15 years | 0.397 | 0.032 | 0.578 | 0.028 |
|  | (0.686) |  | (1.058) |  |
| >15 years | -0.004 | 0.000 | -0.453 | -0.034 |
|  | (0.662) |  | (0.976) |  |
| ***Legal Status*** (Base: sole proprietorship) | |  |  |  |
| Partnership | 0.993 | 0.063 | -0.732 | -0.056 |
|  | (0.755) |  | (0.608) |  |
| LLP | 0.309 | 0.025 | 0.417 | 0.020 |
|  | (1.598) |  | (1.604) |  |
| LTD Co. | 0.251 | 0.020 | -0.309 | -0.020 |
|  | (0.382) |  | (0.579) |  |
| ***Industry*** (Base: Agriculture) |  |  |  |  |
| Manufacturing | -0.198 | -0.024 | -0.069 | -0.005 |
|  | (0.673) |  | (0.769) |  |
| Construction | 0.514 | 0.051 | 0.216 | 0.014 |
|  | (0.618) |  | (0.680) |  |
| Wholesale/retail | 0.876 | 0.078 | -0.346 | -0.029 |
|  | (0.668) |  | (0.690) |  |
| Hotel & catering | 1.410* | 0.106 | 0.008 | 0.001 |
|  | (0.737) |  | (0.774) |  |
| Trans & Comms | 0.815 | 0.073 | 0.117 | 0.008 |
|  | (0.708) |  | (0.822) |  |
| Business Service | 0.671 | 0.063 | -0.175 | -0.014 |
|  | (0.672) |  | (0.716) |  |
| Health | 0.325 | 0.034 | -0.648 | -0.059 |
|  | (0.852) |  | (0.970) |  |
| Other services | 0.970 | 0.083 | 0.431 | 0.027 |
|  | (0.622) |  | (0.708) |  |
| ***Region*** (Base: Scotland) |  |  |  |  |
| North East | -0.383 | -0.029 | 0.401 | 0.038 |
|  | (0.669) |  | (0.791) |  |
| York & Humber | -0.400 | -0.031 | 0.717 | 0.061 |
|  | (0.531) |  | (0.599) |  |
| North West | 0.736 | 0.038 | 0.738 | 0.063 |
|  | (0.697) |  | (0.617) |  |
| West Midlands | -0.594 | -0.049 | 0.883 | 0.071 |
|  | (0.521) |  | (0.658) |  |
| East Midlands | 0.207 | 0.013 | 1.745* | 0.106 |
|  | (0.597) |  | (0.925) |  |
| East England | -0.631 | -0.053 | 0.398 | 0.038 |
|  | (0.668) |  | (0.787) |  |
| Wales | 0.528 | 0.029 | 0.714 | 0.061 |
|  | (0.704) |  | (0.627) |  |
| South West | -0.288 | -0.021 | 0.060 | 0.006 |
|  | (0.617) |  | (0.629) |  |
| London | 0.040 | 0.003 | 1.129* | 0.084 |
|  | (0.567) |  | (0.594) |  |
| South East | -0.339 | -0.026 | 0.119 | 0.012 |
|  | (0.538) |  | (0.527) |  |
| N.Ireland | -0.694 | -0.060 | 0.006 | 0.001 |
|  | (0.730) |  | (0.733) |  |
| *PROFIT* | 0.358 | 0.027 | 0.529 | 0.038 |
|  | (0.280) |  | (0.347) |  |
| ***Risk Rating*** (Base: Minimum) |  |  |  |  |
| Low | 0.859* | 0.073 | 0.922* | 0.079 |
|  | (0.478) |  | (0.486) |  |
| Average | 0.632 | 0.058 | 0.614 | 0.059 |
|  | (0.478) |  | (0.533) |  |
| Above Average | 0.714 | 0.064 | 0.549 | 0.053 |
|  | (0.583) |  | (0.680) |  |
| Not Known | 0.273 | 0.028 | 2.740* | 0.135 |
|  | (0.618) |  | (1.491) |  |
| **Inverse Mills Ratios** |  |  |  |  |
| Historical Rejection | 0.663 | 0.050 | 0.160 | 0.012 |
|  | (1.375) |  | (1.528) |  |
| *SOUGHT* | 2.654*** | 0.199 | 0.546 | 0.040 |
|  | (0.800) |  | (0.956) |  |
| *GOT* |  |  | -1.141 | -0.083 |
|  |  |  | (1.318) |  |
| **Constant** | -7.742** |  | 0.642 |  |
|  | (3.488) |  | (4.422) |  |
| N Obs | 30,577 |  | 30,577 |  |
| Censored N | 1026 |  | 867 |  |
| Wald *χ*2 | 71.87*** |  | 37.67 |  |
| Log likelihood | -206.69 |  | -171.77 |  |

** p <* .10; ** *p* < .05; *** *p* < .01. Asymptotic robust standard errors reported. Weights applied.
